# Deformation imaging and rotational mechanics in neonates: a guide to image acquisition, measurement, interpretation, and reference values

**DOI:** 10.1038/s41390-018-0080-2

**Published:** 2018-08-02

**Authors:** Afif El-Khuffash, Ulf Schubert, Philip T. Levy, Eirik Nestaas, Willem P. de Boode, on behalf of the European Special Interest Group ‘Neonatologist Performed Echocardiography’ (NPE), T Austin, K Bohlin, M. C. Bravo, C. R. Breatnach, M Breindahl, E Dempsey, A. M. Groves, S Gupta, B Horsberg Eriksen, P. J. McNamara, Z Molnar, S. R. Rogerson, C. C. Roehr, M Savoia, C. E. Schwarz, A Sehgal, Y Singh, M. G. Slieker, C Tissot, R van der Lee, D van Laere, B van Overmeire, L van Wyk

**Affiliations:** 1 Department of Neonatology, The Rotunda Hospital, Dublin, Ireland; 2 Department of Pediatrics, The Royal College of Surgeons in Ireland, Dublin, Ireland; 3 Department of Clinical Science, Intervention and Technology, Karolinska Institutet, Stockholm, Sweden; 4 Department of Pediatrics, Washington University School of Medicine, Saint Louis, MO USA; 5 Department of Pediatrics, Goryeb Children’s Hospital, Morristown, NJ USA; 6 Institute of Clinical Medicine, Faculty of Medicine, University of Oslo, Oslo, Norway; 7 Department of Cardiology and Center for Cardiological Innovation, Oslo University Hospital, Rikshospitalet, Oslo, Norway; 8 Department of Paediatrics, Vestfold Hospital Trust, Tønsberg, Norway; 9 Department of Neonatology, Radboud University Medical Center, Radboud Institute for Health Sciences, Amalia Children’s Hospital, Nijmegen, The Netherlands; 10 Department of Neonatology, Rosie Hospital, Cambridge University Hospitals NHS Foundation Trust, Cambridge, United Kingdom; 11 Department of Neonatology, Karolinska University Hospital, Karolinska Institutet, Stockholm, Sweden; 12 Department of Neonatology, La Paz University Hospital, Madrid, Spain; 13 Department of Neonatology, The Rotunda Hospital, Dublin, Ireland; 14 Karolinska University Hospital, Karolinska Institutet, Stockholm, Sweden; 15 INFANT Centre, Cork University Maternity Hospital, University College Cork, Cork, Ireland; 16 Division of Newborn Medicine, Mount Sinai Kravis Children’s Hospital, New York, NY USA; 17 University Hospital of North Tees, Durham University, Stockton-on-Tees, United Kingdom; 18 Department of Pediatrics, Møre and Romsdal Hospital Trust, Ålesund, Norway; 19 Departments of Pediatrics and Physiology, University of Toronto, Toronto, ON Canada; 20 John Radcliffe Hospital, Oxford, United Kingdom; 21 The Royal Women′s Hospital, Parkville, VIC Australia; 22 Department of Paediatrics, University of Oxford, John Radcliffe Hospital, Oxford, United Kingdom; 23 Azienda Ospedaliero-Universitaria S. Maria della Misericordia, Udine, Italy; 24 Department of Neonatology, University Children’s Hospital of Tübingen, Tübingen, Germany; 25 Department of Pediatrics, Monash University, Melbourne, Australia; 26 Addenbrooke′s Hospital, Cambridge University Hospitals NHS Foundation Trust, Cambridge, United Kingdom; 27 Department of Paediatric Cardiology, Radboudumc Amalia Children’s Hospital, Nijmegen, The Netherlands; 28 Department of Pediatrics, Clinique des Grangettes, Chêne Bougeries, Switzerland; 29 Department of Neonatology, Radboud university medical center, Radboud Institute for Health Sciences, Amalia Children’s Hospital, Nijmegen, The Netherlands; 30 Department of Pediatrics, Antwerp University Hospital UZA, Edegem, Belgium; 31 Department of Neonatology, University Hospital Brussels, Brussels, Belgium; 32 Department of Paediatrics & Child Health, University of Stellenbosch, Cape Town, South Africa

## Abstract

Advances in neonatal cardiac imaging permit a more comprehensive assessment of myocardial performance in neonates that could not be previously obtained with conventional imaging. Myocardial deformation analysis is an emerging quantitative echocardiographic technique to characterize global and regional ventricular function in neonates. Cardiac strain is a measure of tissue deformation and strain rate is the rate at which deformation occurs. These measurements are obtained in neonates using tissue Doppler imaging (TDI) or two-dimensional speckle tracking echocardiography (STE). There is an expanding body of literature describing longitudinal reference ranges and maturational patterns of strain values in term and preterm infants. A thorough understanding of deformation principles, the technical aspects, and clinical applicability is a prerequisite for its routine clinical use in neonates. This review explains the fundamental concepts of deformation imaging in the term and preterm population, describes in a comparative manner the two major deformation imaging methods, provides a practical guide to the acquisition and interpretation of data, and discusses their recognized and developing clinical applications in neonates.

## Introduction

Characterization of myocardial adaptation with echocardiography during the critical periods of development in neonates is important for recognition and management of circulatory disturbances.^1^ Conventional measures of left ventricular (LV) function, such as shortening (SF) and ejection fraction (EF), assess the changes in cavity dimensions, but are insufficient to detect overt dysfunction in a timely manner, because their measurements are influenced by image quality and inadequate reproducibility and standardization in neonates.^2^ Similarly, conventional techniques to estimate right ventricular (RV) performance rely largely on quantitative estimates and qualitative predictions that are limited because of the unique three-dimensional structure of the chamber.^3,4^ Although conventional echocardiography with tissue Doppler (TD) velocity is considered to be reliable for ventricular wall motion analysis, the visual estimation of wall motion is very subjective and cannot be utilized as an aid to clinical assessment.^5^ Many challenges also exist for serially monitoring the cardiovascular status of preterm infants and sick term infants, largely due to the relative insensitivity of clinical markers (such as blood pressure and capillary refill time) in defining hemodynamic compromise.^6,7^ For the management of a broad range of heart diseases, a quantitative, reproducible approach for the serial assessment of cardiac function is of paramount importance in neonates.^8^

The concept of deformation imaging is a novel technique, recently introduced to the field of neonatology, and can be measured by either speckle tracking echocardiography (STE) or tissue Doppler imaging (TDI).^9–11^ Myocardial strain (*ε*) is a measure of tissue deformation and strain rate (SR) is the rate at which deformation occurs. Both are feasible and reproducible markers of global and regional performance that provide fundamental information on myocardial properties and mechanics that would otherwise be unavailable with conventional imaging.^3,9–12^ Myocardial strain can be measured in terms of three normal strains (longitudinal, radial, and circumferential) and six shear strains.^13^ Currently, only normal strain and shear strain in the circumferential-longitudinal plane (rotational mechanics) have been investigated for clinical use in neonates.^9^ A thorough understanding of the basic principles of deformation imaging, recognition of their applicability in term and preterm infants, strengths, and limitations is essential for advancing those techniques to routine clinical care. This review explains the fundamental concepts of deformation imaging, describes in a comparative manner the two major deformation imaging methods, provides a practical guide to the acquisition and interpretation of data, and discusses the clinical applications and available reference ranges in the term and preterm population.

## Basic concepts of myocardial deformation

**Fig. 1:**
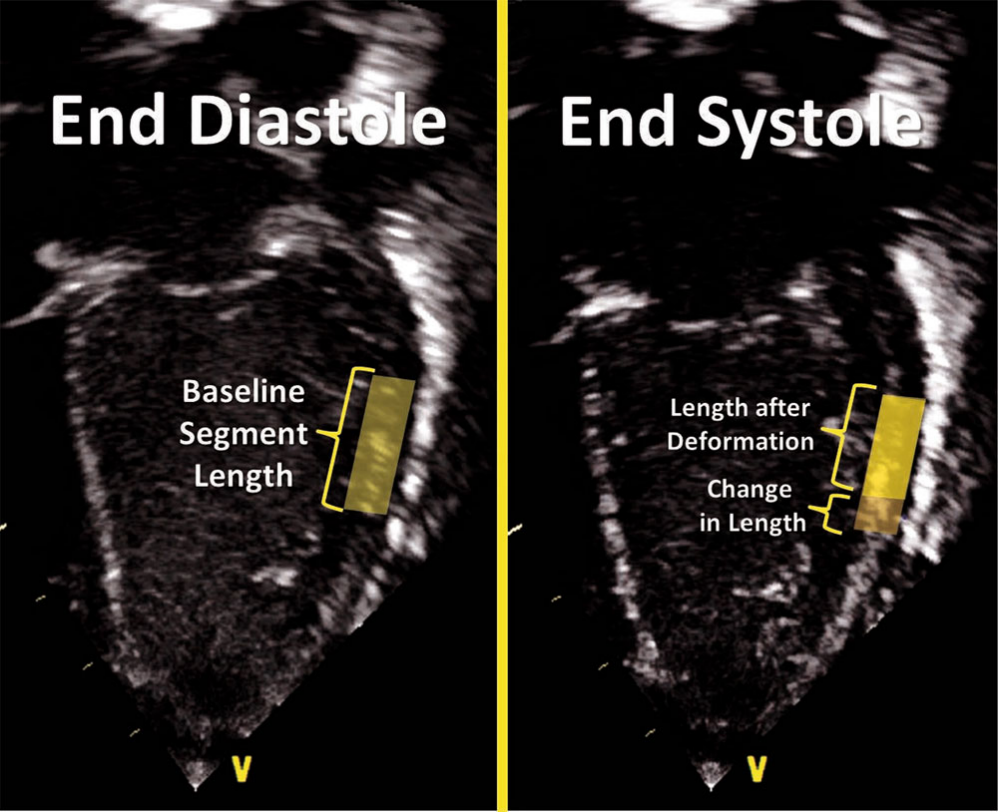
Principles of deformation. Longitudinal strain refers to the change in length of a segment from its baseline length in end-diastole to its deformed shape in systole. Strain refers to the degree of change in shape relative to the baseline and is expressed in %. Shortening reflects negative values and lengthening positive values. In this image, shortening of the mid-segment of the LV free wall is illustrated

**Fig. 2:**
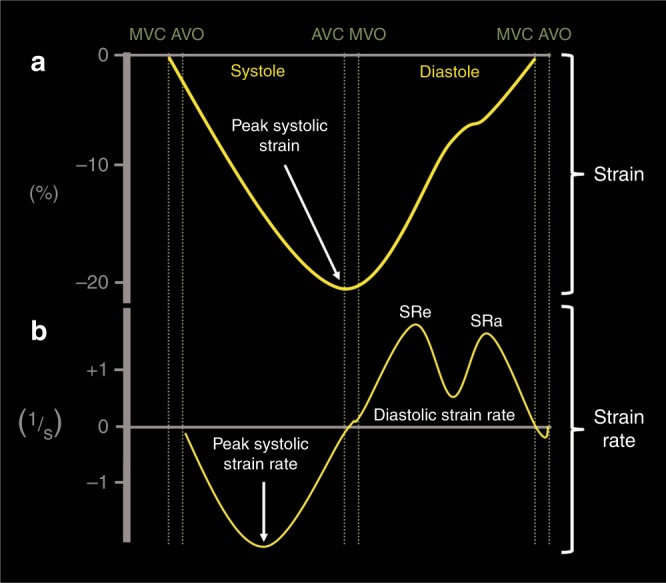
Strain and strain curves in one cardiac cycle in the ventricle. **a** Strain usually peaks during end-systole at aortic valve closure (AVC) and returns to baseline during diastole at mitral valve closure (MVC). **b** Strain rate usually peaks in mid-systole and returns to baseline at AVC when no deformation occurs. During diastole, the rate of strain returning to baseline is biphasic. MVO mitral valve opening, AVO aortic valve closure, SRe early diastolic strain rate, SRa late diastolic strain rate (during atrial contraction)

Deformation refers to the change in the shape of the myocardium from its baseline shape at end-diastole to its deformed shape at end-systole.^9^ This occurs in response to sarcomere shortening due to contraction (Fig. 1). The deformation leads to a reduction in cavity size and ejection of blood from the ventricle. Myocardial strain (*ε*) is a dimensionless index that is defined as the relative tissue deformation under an applied force, and is expressed as a percentage (%; see Geyer et al.^14^). SR (s^−1^) is the first derivative of strain, or the speed at which deformation occurs in systole.^14^ When applied to the neonatal myocardium, strain and SR are used as measures of global and regional ventricular function (Figs. 2 and 9).

Myocardial strain can be measured in terms of “normal” strain and “shear” strain.^13^ Normal strain is caused by forces that act perpendicular to the surface of the myocardial wall, resulting in stretching or contraction without skewing of the volume.^15^ There are three types of normal strain; longitudinal, radial, and circumferential. Conversely, forces causing shear strain act parallel to the surface of the wall and lead to a shift of volume borders relative to one another as delineated by a “shear” angle.^15^ There are six forms of shear strain grouped into three categories; circumferential-longitudinal, circumferential-radial, and longitudinal-radial. Myocardial shear in the circumferential-longitudinal plane results in twist or torsional deformation of the LV during ejection. Only the three normal forms of strain and circumferential-longitudinal shear strain (rotational mechanics) have been investigated for clinical use in neonates.^9^

## Methods of deformation assessment

**Fig. 3:**
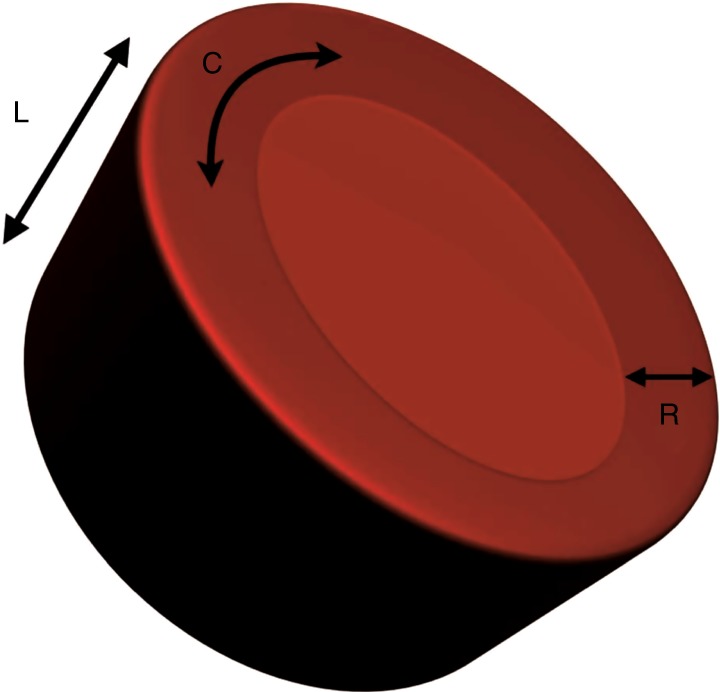
LV deformation. LV deformation occurring in three directions; L longitudinal, C circumferential, R radial

LV and RV deformation patterns differ based on their own unique myoarchitectural fiber orientation. The LV myocardium consists of circumferential fibers in the midwall layer and longitudinal fibers in the endocardial and epicardial layers.^16^ During myocardial contraction, the LV wall shortens and thickens with LV deformation occurring in three directions: (i) longitudinal shortening that is directed from base to apex in the apical four-chamber view, (ii) circumferential shortening along the circular perimeter observed in a parasternal short-axis view, and (iii) radial thickening directed toward the center of the LV cavity measured in the parasternal short axis (^14^; Fig. 3). Deformation is assigned a negative sign for shortening (in longitudinal and circumferential planes) and a positive sign for thickening in the radial plane.

In the circumferential-longitudinal plane, the net difference in the systolic rotation of the myocardium between the apical and basal short-axis plane is referred to as twist (measured in degrees) and represents the wringing motion of the LV during systole. If normalized to the distance between the respective image planes, it is referred to as torsion (°/cm). LV rotational mechanics (twist and torsion) are assessed by STE.

Compared with the LV, the RV myofiber architecture is composed of superficial oblique and dominant deep longitudinal layers. The myofibers in the RV are aligned in a more longitudinal direction than in the LV, and as the dominant pattern of RV deformation, longitudinal shortening provides the major contribution to stroke volume during systole and is a more sensitive indicator of RV dysfunction.^3,13,17^ Deformation in the circumferential and radial directions in the RV may prove to be a valuable measure of function in certain neonatal conditions (i.e., congenital heart disease), but there is a paucity of studies that use these measures in clinical practice and those studies have not been able to demonstrate significant reliability in neonates.^18,19^

There are two established methods for assessing and calculating deformation entitled *Lagrangian strain* and *Eulerian (natural) strain*.^14,20^ *Lagrangian strain* refers to the change in length relative to an unstressed baseline length, against which all subsequent deformation will be measured.^21^ Since Lagrangian strain is measured as the separation distance between two regions of myocardium relative to the original separation distance in end-diastole, it is not affected by the heart rate (HR).^22^ STE lends itself more readily to the calculation of Lagrangian strain, since the baseline length is always known and can easily be used as a reference.^14^ *Eulerian strain* calculation is based on a reference length that is different at each interrogation time point, i.e., each color TD frame, and is better suited for use with TDI. Natural and Lagrangian strains are related so that one can be converted into the other. STE software packages will report Lagrangian strain, but natural strain (i.e., TD) can be derived from STE by conversion from the Lagrangian strain. Studies that utilized strain and SR measures to characterize ventricular function in neonates must therefore indicate the software package and the type of strain or SR.

## Influences of myocardial contractility and load dependence

**Fig. 4:**
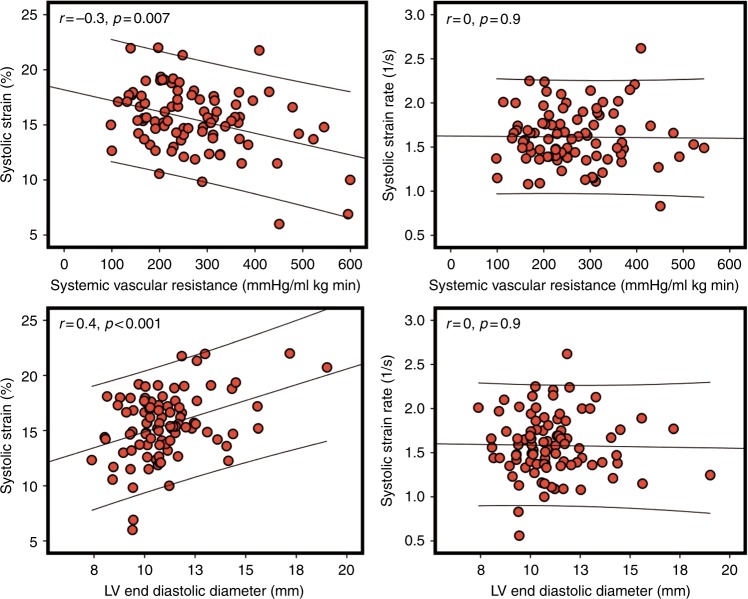
The relationship between loading conditions and deformation parameters. There is a negative relationship between strain and systemic vascular resistance (a surrogate of afterload) but a positive relationship between strain and left ventricle end-diastolic diameter (a surrogate of preload). Note the lack of relationship between systolic strain rate and loading measures (data set from ref. ^26^)

Deformation is affected by several factors that should be considered when using strain imaging in clinical practice. Specifically, global and regional strains (%) are influenced by preload (which increases wall strain) and afterload (which reduces wall strain; ^14^). Compared to strain, SR is thought to be less dependent on loading conditions, and is a more accurate reflection of intrinsic myocardial contractile function.^23^ Preclinical studies in animal models have demonstrated that preload has a positive impact on strain, whereas increasing afterload is associated with its reduction.^23–25^ In preterm clinical studies, surgical ligation of patent ductus arteriosus (PDA) results in a sudden elevation in LV afterload and a reduction in LV preload that significantly decreases LV strain in the immediate post-operative period.^2^ In the early transitional period in preterm infants, there is a negative correlation between strain and measures of afterload and positive correlation between strain and measures of preload.^19,26^ Antenatal magnesium sulfate administration is associated with lower systemic vascular resistance (SVR) and higher myocardial function as measured by strain imaging, but SR appeared to be less influenced by loading conditions, further validating the lower SR dependency on loading conditions (^27^; Fig. 4).

## Variability and standardization of deformation imaging

Three factors modulate variability in deformation imaging; these include variability in image acquisition, intra- and inter-observer variability in post-acquisition processing, and differences between echocardiographic equipment and proprietary software for image analysis.^3,28,29^ This review addresses the first two factors (proper image acquisition and validation of deformation measurements), but in light of the push to standardize the acquisition of these measures and reduce inter-vendor differences and ambiguities,^21,30^ it is important for the reader to review any details of hardware settings, manual settings, and local imaging protocols to get a better understanding of the values presented.

To measure and calculate myocardial strain and SR, there are two different types of echocardiographic imaging modalities: TDI and STE. First introduced as a post-processing feature of TDI with velocity data converted to strain and SR, strain imaging information has more recently also been derived from STE computer processing.^31^ These imaging modalities derive information on myocardial strain and SR from two fundamentally different ways and will be considered separately.

## Tissue doppler deformation imaging

### Principles and validation in neonates

**Fig. 5:**
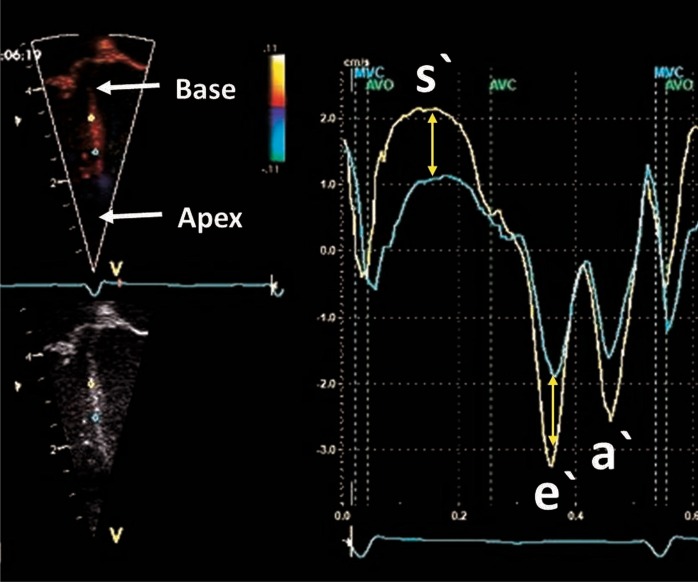
Difference in velocity between two points along the long axis of the septum. The curves show tissue velocities by tissue Doppler during the cardiac cycle. The point closer to the base (yellow) has a higher systolic and diastolic velocity when compared with the point closer to the base (green). The difference in velocity is used to calculate strain rate and derive strain of that segment bordered by the two points

Echocardiographic strain was first derived from TDI velocity data using the Doppler equation to convert ultrasound frequency shifts to velocity information along the scan lines.^31^ In the longitudinal plane of the ventricle, there is a velocity gradient from the base of the heart toward the apex. Basal myocardial tissue moves at a higher velocity (toward the apex in systole) than myocardial tissue at the apex due to the tethering effects and the stationary position of the apex (Fig. 5). TD-derived (longitudinal) deformation imaging calculates SR by assessing the difference in velocity (the velocity gradient) between points along the longitudinal plane. Strain is then assessed by integrating the SR values by time. Only velocities along (parallel to) the beam of the ultrasound are measured by the TD method; therefore, deformation indices measured using TD are highly dependent on the angle of insonation. Due to the high temporal resolution of this technique, TD is well suited for measurement of SR values in neonates (with a higher baseline HR) as it employs a calculation method that utilizes the high TD frame rates (FRs) (>180 frames per second; ^26,32^). In neonates, TDI-derived deformation values can be obtained from several regions of the heart. Longitudinal deformation can be measured from most parts of the LV and RV and is more often measured in neonates.^26,32^ Circumferential and radial deformation can only be assessed in a few LV regions.^33^

To characterize ventricular function with longitudinal strain, some studies have reported values from many heart segments,^33–38^ while others have obtained values from the basal segment of the LV and RV free walls, in addition to the septum.^26,39–41^ The LV base remains the most challenging segment to assess with TD. The reliability of the results obtained from the LV base is reduced because artifacts arising from the lungs often obscure the base. This high dependency on the requirement for clear imaging of the myocardial walls and the angle dependency (it is difficult to maintain an angle insonation less than 20° between the ultrasound beam and the LV wall), coupled with the artifacts of extra-cardiac structures and image dropouts, can lead to over- and underestimation of strain and SR values.^42^

The feasibility and reproducibility of TD-derived deformation parameters have been established in term and preterm neonates.^11,36,37,41^ The most reproducible measurements are assessed in longer segments and when the LV base free wall is avoided as site of measurement.^35^ Nestaas et al. conducted the first studies of reproducibility in term infants and revealed moderate reproducibility in obtaining strain and systolic SR with intraclass correlation coefficients (ICC) ranging between 0.6–0.7 for intra-observer and 0.4–0.5 for inter-observer repeated measures.^36,43^ With enhanced image optimization techniques, reliability data have improved in term neonates for basal strain with coefficients of variation (COV) values <15% and ICC values >0.75.^35,38,40^ In preterm infants, measurements are feasible in about 90% of studies when adequate imaging quality is achieved.^41^ Poon et al. reported COV values of <5% for LV, septal and RV basal strain in preterm infants.^38^ Helfer et al. further illustrated more modest reproducibility results in preterm infants; the septum showed the best COV ranging from 10% to 34% for intra- and 22% to 27% for inter-observer variability, whereas the left wall had a higher and wider range of values (17–57% for intra- and 29–60% for inter-observer variability; ^39^). James et al. demonstrated that reproducibility of basal longitudinal strain and SR measurements in the RV and septum were more favorable than the LV in preterm infants.^41^ All of these studies in preterm and term infants consistently show reduced reproducibility of LV basal strain values, and cited poor image quality and difficulty in obtaining an angle on insonation <20° as the main reasons.

### Image acquisition and offline data measurement

To obtain reliable deformation values, strain by TD imaging and post-processing analysis protocols have been developed and implemented in neonates.^36,43^ A clear electrocardiogram (ECG) signal with a well-defined QRS complex is necessary for obtaining a complete cardiac cycle for offline processing. Pulsed wave Doppler of the aortic and mitral valves should be used to annotate the timing of events. Timing of the aortic valve closure may also be obtained from the TDI curves.^44^ An apical four-chamber view is used to obtain a clear image of the walls with minimal artifact. The transducer should be manipulated to align the wall of interest parallel to the ultrasound beam. The sector width and depth should be narrowed to just beyond the borders of the wall to obtain a high FR (>180). The velocity scale (pulse repetition frequency, PRF) should be adjusted to avoid aliasing. Three-cycle analysis is more reproducible than single-cycle analysis in TD imaging, and a minimum of three cycles must be recorded for offline processing.^1^

**Fig. 6:**
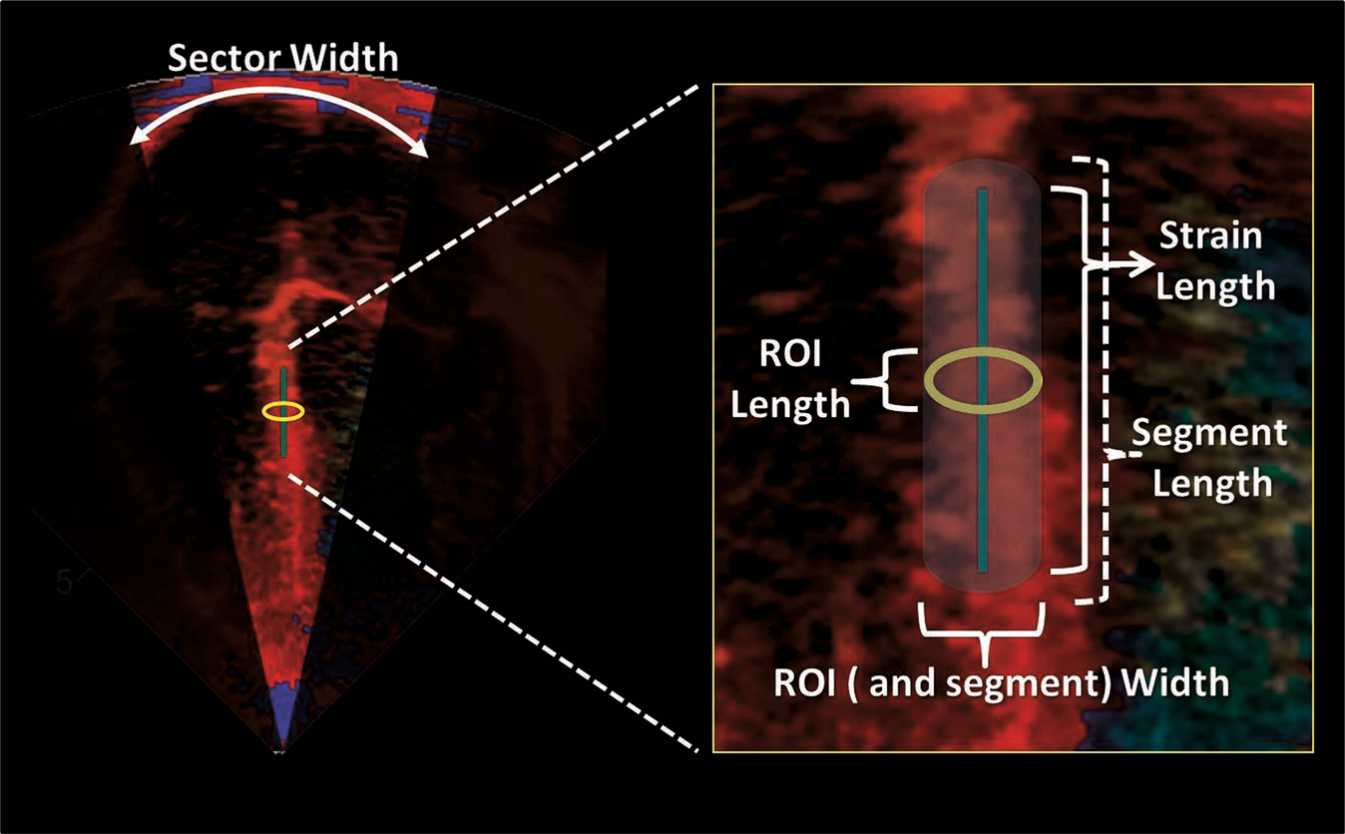
Offline measurement of SR and strain using tissue Doppler. The sector width should be narrowed to increase the frame rate. The basal segment of the wall is usually interrogated to obtain SR and strain values. The ROI dimensions (length and width) are set by the operator. Strain length is also set while ensuring that the borders of the segment are not in contact with artifact or atrial tissue. The ROI can be moved slightly along the wall to obtain a clean and noise-free SR and strain curve (see Fig. 7)

**Fig. 7:**
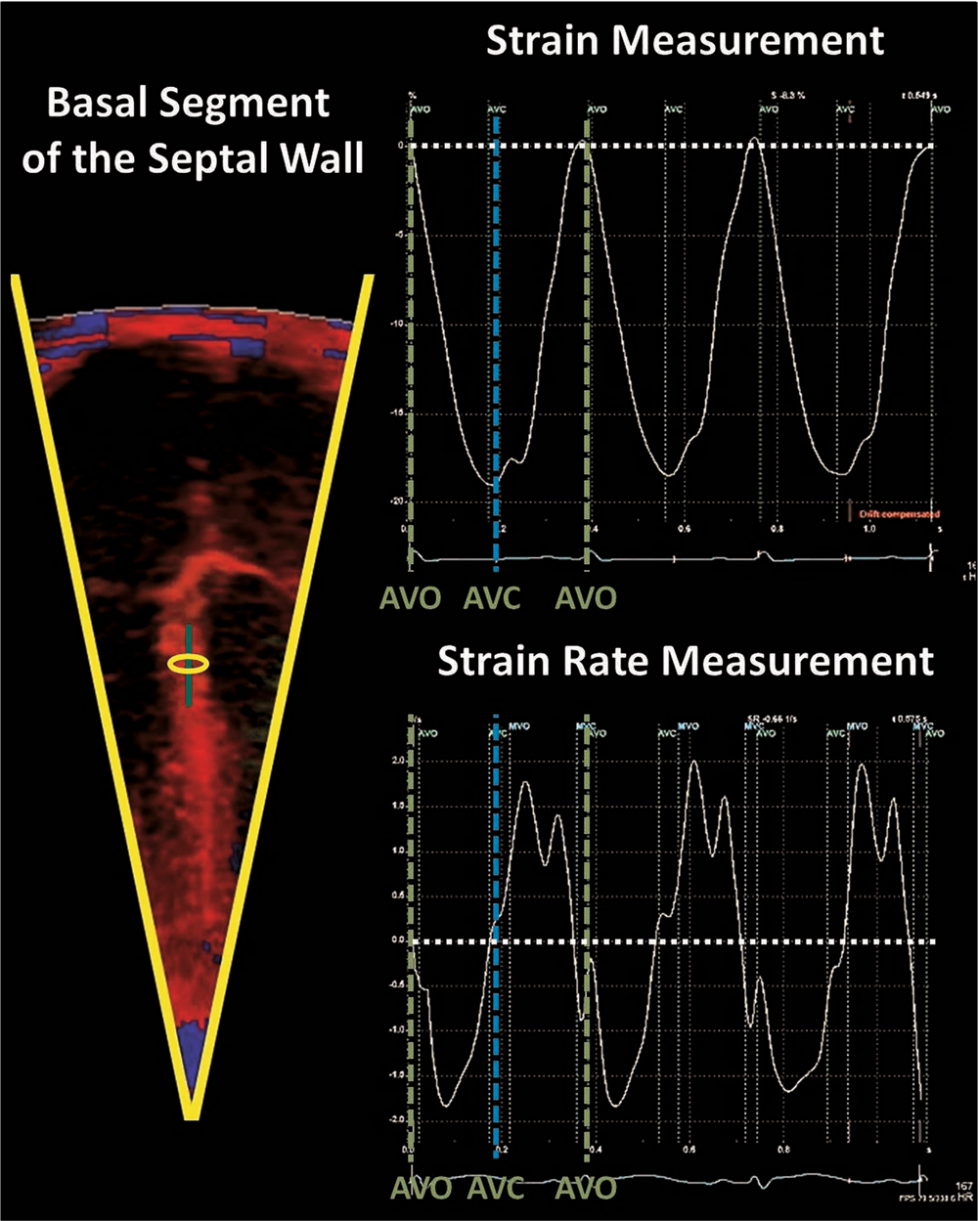
An example of clear and artifact-free strain and strain rate curves over three cardiac cycles. Note the timing of events within the cardiac cycle. Strain peaks at end-systolic at aortic valve closure (AVC) and systolic strain rate peaks in mid-systole between aortic valve opening (AVO) and AVC

Strain and SR values are generated during offline analysis. The parameters are derived from a sample area (segment). The size of the segment is set by the size of a specific region of interest (ROI) within the myocardial wall, which is determined by the operator (length and width) and a strain length (SL). The length of the ROI should be adequate for optimal calculation, while minimizing noise, and the width should not be larger than the width of the actual wall of interest. The operator must set the SL, also referred to as a computational distance. The SL is the length along the ultrasound beam against which the velocities for each point within the ROI are compared to derive the velocity gradient. The segment size will be larger than the ROI as it stretches parallel to the ultrasound beams toward the apex and the base of the heart. The SL should not project outside the borders of the wall (into the atrial tissue for example; Fig. 6). Additional “optional” settings, i.e., Gaussian smoothing and drift compensation, can be utilized to minimize noise; however, with good image quality these settings are most likely not necessary (Fig. 7). Optimal probe choices, ideal ROI width and length and SL lengths have been published for term and preterm infants and are summarized in Table 1.^35,36,38,39,43^

**Table 1:** Optimal setting for tissue Doppler deformation measurement

	Term infants	Preterm infants
Probe	5s or 7s	10s or 12s
Sector width	Narrow	Narrow
Sector depth	Shallow	Shallow
Velocity scale (cm/s) (avoid aliasing)	−16 to 16	−16 to 16
Transducer frequency (MHz)	2.5–3.0	8.0
Pulse repetition frequency (kHz)	1.0	2.0
Velocity scale (cm/s)	16	16
Frame rate (FPS)	>200	>200
Region of interest length (mm)	1	1
Region of interest width (mm)	2 or 3	2 or 3
Strain length (mm)	10–20	6
Linear drift compensation	On	On
Gaussian smoothing	On	On

These settings apply for the General Electric Vivid scanners and Echo Pac Software (GE Medical, Milwaukee, USA). These recommendations are a guide only and setting may differ depending on new information emerging

### Reference ranges and clinical applicability in the neonatal population

Reference ranges for deformation parameters exist in the term and preterm neonates^26,35,37,45^ (Table 2). In the term population, LV longitudinal strain values range between −20 and −25 (%), while SRs values range between −1.5 and −2.5 1/s, SRe between 2.8 and 3.2 1/s, and SRa slightly lower between 2.1 and 2.4 1/s. The RV free wall has higher strain values when compared with LV free wall and septum; however, SR values are comparable. The differences between LV and RV strain values may reflect the differing loading conditions between the RV and LV in the early neonatal period that may have an impact on strain but not SR. HR and the persistence of fetal shunts during the early transitional period appear to have a negligible impact on the measurements, but age may play a role in the first year of age.^11^

**Table 2:**
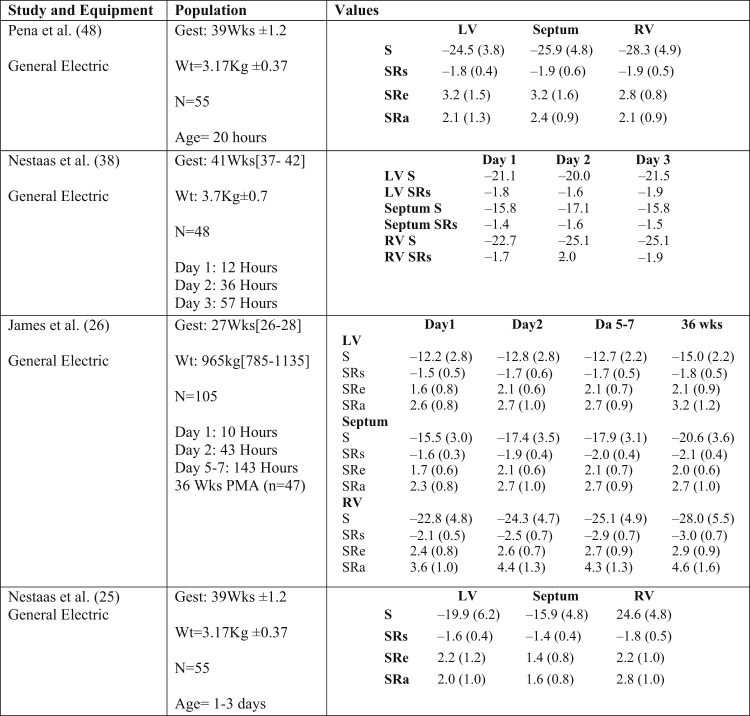
Reference ranges for tissue Doppler deformation parameters in the neonatal population

Values presented as means and standard deviations if available. All values are obtained from the basal segments in the four-chamber view. All strain units are %, strain rate 1/s

*Gest* gestation, *N* number of infants, *Wt* birthweight, *Wks* weeks, *LV* left ventricle, *RV* right ventricle, *PMA* post-menstrual age, *S* strain, *SRs* systolic strain rate, *SRe* early diastolic strain rate, *SRa* late diastolic strain rate (during atrial contraction)

There is early evidence of the utility of deformation parameters in several disease states in term infants. Nestaas et al. demonstrated that LV and RV deformation parameters are uniformly lower in infants with hypoxic ischemic encephalopathy (HIE) compared with healthy controls.^35^ Interestingly, these differences occurred while SF was preserved between groups, further demonstrating that strain imaging permits a more comprehensive assessment of myocardial performance in neonates that could not be previously obtained with conventional imaging.^35^ The same group of investigators also demonstrated that infants with HIE have similarly impaired myocardial function during days 1–3, irrespective of whether they received therapeutic hypothermia, suggesting that myocardial injury may be a result of the initial insult rather than “cooling” treatment.^46^ In term infants with severe persistent pulmonary hypertension of the newborn (PPHN) not responsive to inhaled nitric oxide, RV strain significantly improves following the administration of milrinone over a 24-h period.^47^ With the establishment of reference values, TD-derived deformation measures can now be used to assess the efficacy of patient management strategies in health and disease and monitor treatment response.

In the preterm population, transitional strain and SR values are uniformly lower than those of term infants (Table 2). Two studies have examined the maturational changes of basal deformation parameters over the first few weeks of age.^26,34^ LV free wall strain and SR values remain stable over the first week of age with LV global strain showing a slight increase by 36 weeks post-menstrual age (PMA). Septal and RV free wall show a steadier increase over the first week of age and through 36 weeks PMA.^26,34^ Like term infants, weight, gestation, and HR have a minimal impact on these parameters. However, in the early transitional period, there is a negative correlation between echocardiography-derived estimates of SVR and LV and septal strain values, and a positive correlation between increasing preload associated with a PDA and LV strain.^26,34^ The relationship between SR values and cardiac loading measures were less pronounced, further supporting the dominant load dependency of strain but not SR. Finally, infants with chronic lung disease (CLD) have lower RV strain (–26.4 vs. –30.7%, *p* = 0.0.1) and RV SRa [4.2 vs. 5.3 1/s, *p* = 0.04) independent of gestation. CLD was shown to be associated with increased pulmonary arterial pressure, which may explain this association.^34^

## Two-dimensional SPECKLE TRACKING ECHOCARDIOGRAPHY

### Principles and validation in the neonatal population

Two-dimensional STE (2D STE) is an imaging technique that uses standard B-mode images to measure deformation by tracking the movement of speckles within the myocardial wall. Speckles represent fixed tissue markers, or “natural acoustic markers,” that are randomly distributed throughout the myocardium and have their own unique signature or “fingerprint”.^20^ The speckle patterns result from acoustic backscatter generated by the reflected ultrasound beam. The movement of this speckled pattern follows myocardial tissue motion as it tracks the defined region of speckles, frame by frame and eventually over the entire heart cycle deriving the following information from segments of the myocardial wall: displacement (the movement of those speckles), velocity (the speed at which this movement occurs), strain (the relative change in distance between those speckles), and SR (the speed at which the change in distance occurs; ^9^).

**Fig. 8:**
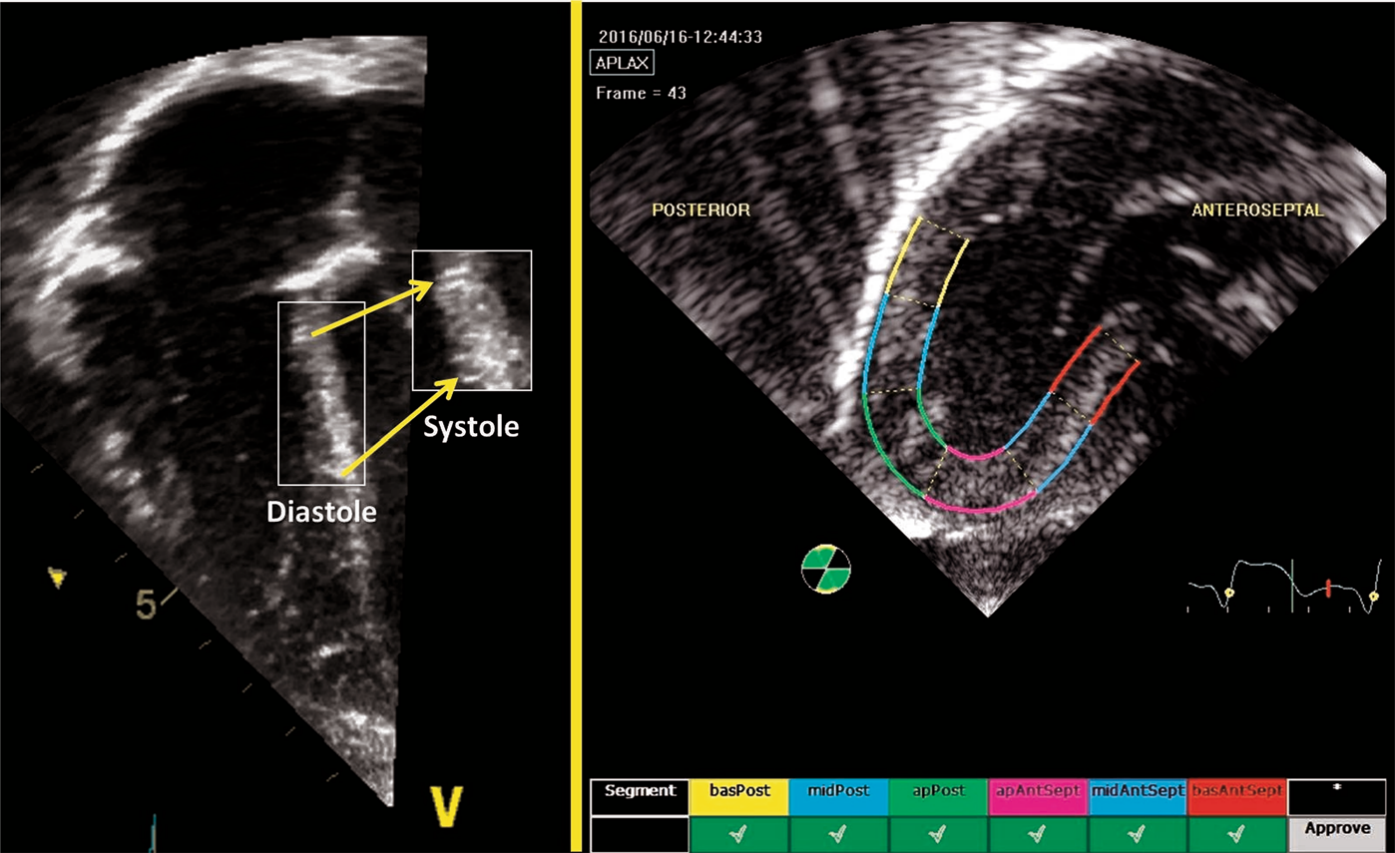
2D Speckle Tracking Echocardiography. Speckles are acoustic back scatter that form a unique pattern within the myocardial walls. Those can be tracked throughout the cardiac cycle to derive deformation measurements. In this apical 3-chamber view of the LV, the myocardial walls are divided into segments and deformation parameters are presented individually for each segment to determine regional function. In addition, deformation for the whole region of interest is used to determine global function

Specialized measuring software programs divide the myocardial walls of interest into segments and generate strain and strain rate values for each region (Fig. 8). Both regional and global functional parameters can be derived using this 2D STE deformation. 2D STE is angle independent within the ultrasound sector allowing the software to track the speckles in any direction.^48^ This angle-independency is the major advantage of 2D STE over TD-derived deformation, as the alignment of the wall relative to ultrasound beam is not necessary. With this relative freedom, enhanced imaging of the myocardial walls is possible. This is of particular importance to the LV free wall, as it can now be imaged at an angle to avoid lung artifact. However, 2D STE employs relatively lower FRs than TD-derived deformation (80–120 frames/s vs. >200 frames/s) and SR parameters, which rely on high temporal resolution, may not be as easy to interpret as strain, particularly in preterm infants with high HRs. Circumferential deformation is more prone to under sampling due to low FRs when compared with longitudinal deformation;^49^ generally, under sampling is avoided by a FR/HR ratio above 1 frame per second/beat per minute (in vitro model). Although 2D STE is less influenced by artifact, it remains highly reliant on clear imaging of the walls without dropouts.^50^

2D STE strain imaging is feasible in neonates with over 85% of acquisitions deemed as adequate quality to analyze.^2,3,11,32^ In preterm infants, the reproducibility of LV global longitudinal strain (LV GLS) is high with intra- and inter-observer ICC values of 0.92 and 0.93, respectively, and Bland–Altman analysis showing no significant bias between observers, with good agreement.^2,11^ de Waal et al. also reported that LV circumferential strain was highly reproducible with intra- and inter-observer ICC >0.85 and COV <10%, but radial strain demonstrated very poor reproducibility with COV values between 18 and 50%.^19,32^ With RV-focused imaging, there is a high degree of intra-observer (bias 3%; COV 2.7%; ICC 0.97) and inter-observer (bias 7%; COV 3.9%; ICC 0.93; *p* < 0.05) agreement for RV longitudinal strain.^3^ In term infants, Jain et al. demonstrated that LV and RV longitudinal strain measurements are highly reproducible with ICCs >0.9 and COVs <10%.^10,51^ Recently, Nestaas et al. demonstrated that the intra- and inter-observer ICC for longitudinal peak systolic strain and SR were all above 0.87 for LV and RV analysis.^1^ Septal strain and SR from a nine-segment model of the three apical views also have moderate reproducibility measures.^11^

### Image acquisition and offline measurement

**Fig. 9:**
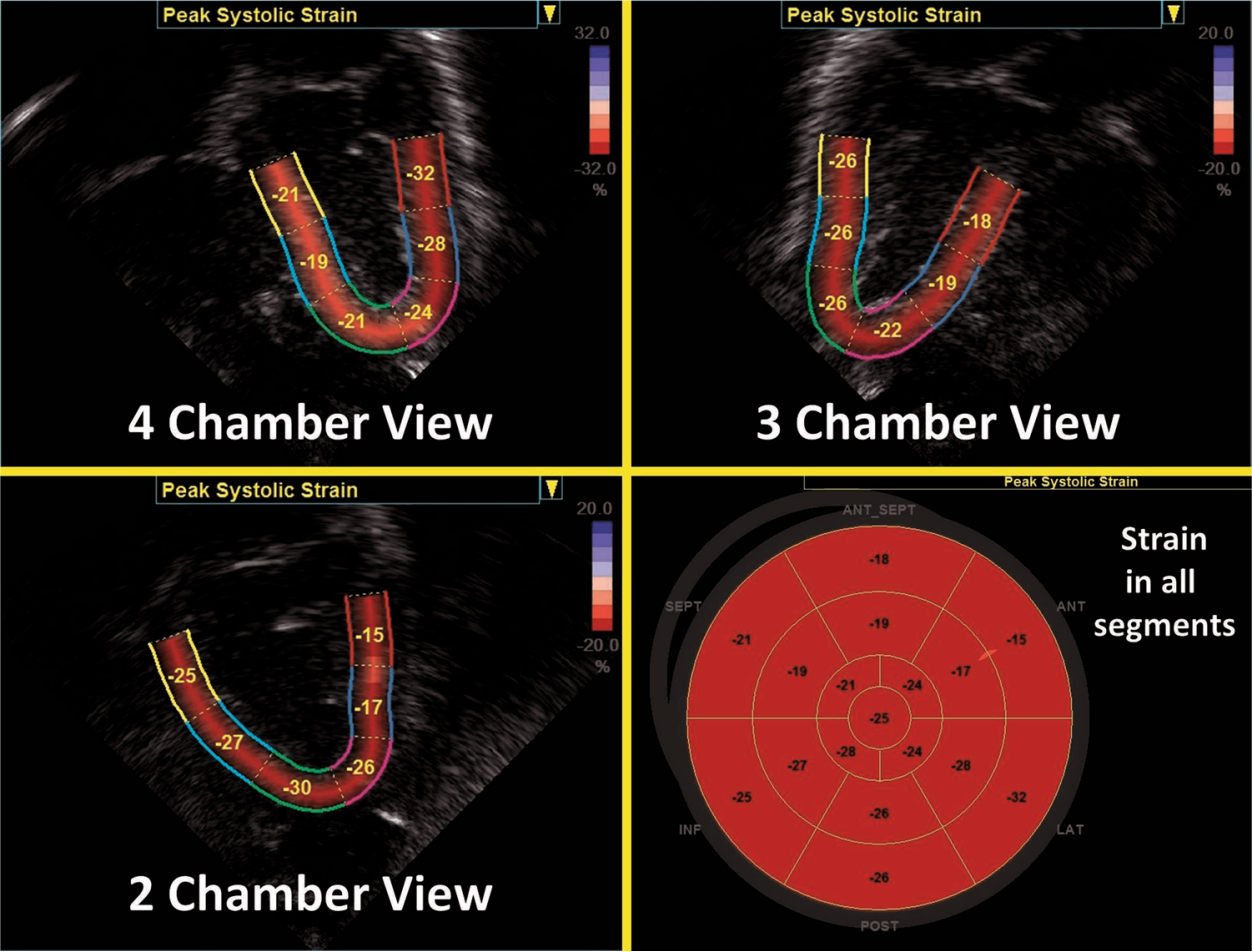
Segmental strain in the three apical planes of the LV and a summary in a “Bullseye” pattern. Global longitudinal strain (often referred to as GLS) is the peak value in a compound curve made from the region of interest from the three planes

**Fig. 10:**
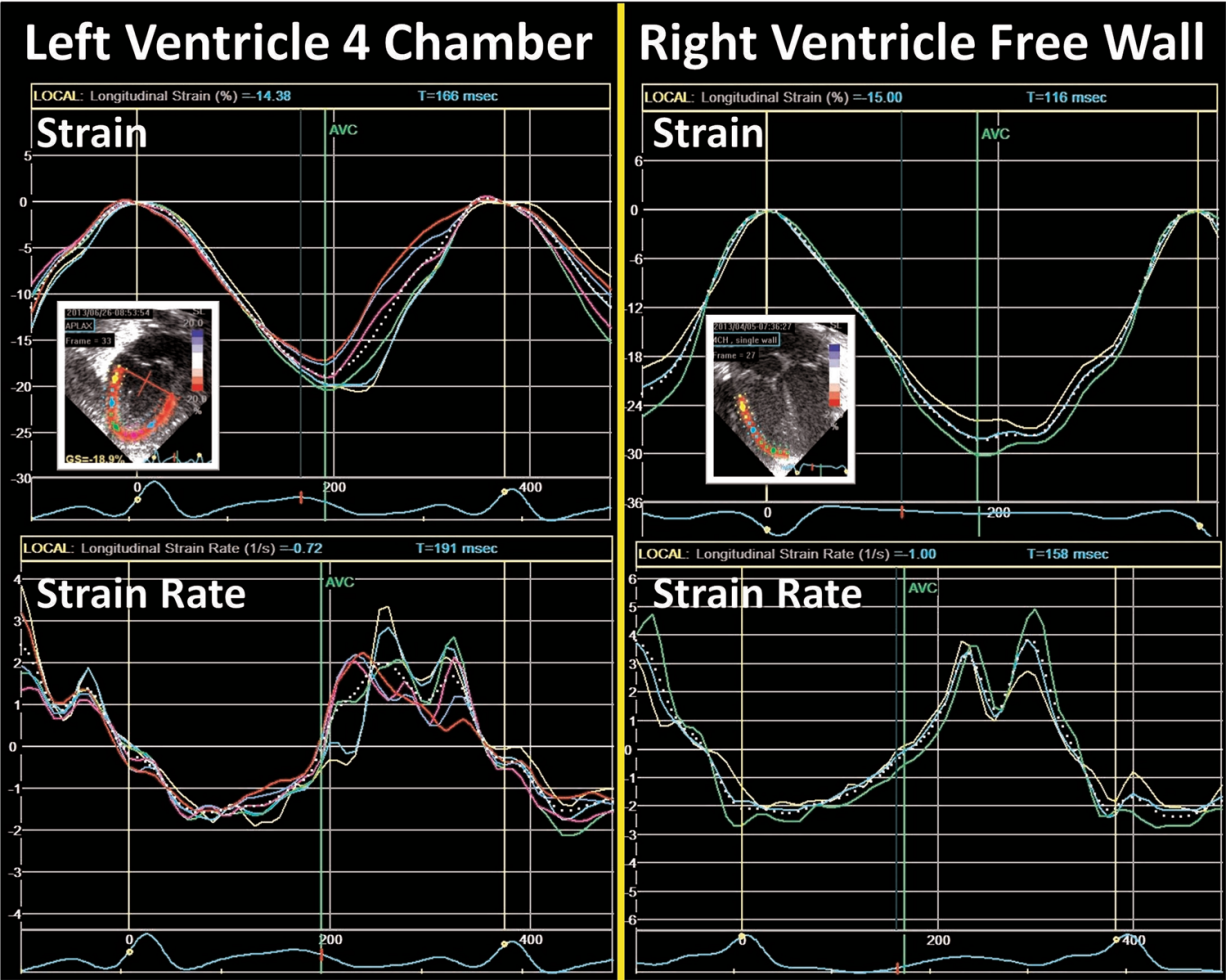
Strain and strain rate curves from the LV four-chamber view and the RV free walls. The colored lines represent the deformation values from each segment and the dotted white line represents the values from the whole region of interest. Notice the relative increased level of noise in the strain rate curves (see text)

Regional and global LV longitudinal deformation parameters are obtained from the apical four-, two-, and three-chamber views (Fig. 9). LV circumferential, radial, and rotational deformation are obtained from the parasternal short axis view at the level of the mitral valve (base), papillary muscles (mid-ventricular), and the apex.^50^ RV longitudinal deformation is obtained from a focused RV four-chamber view (^3,18,52^; Fig. 10), and has been shown to provide the most feasible and reproducible measure of RV longitudinal deformation analysis in neonates.^3^ A modified RV three- chamber view can also be used to obtain RV deformation parameters. This view allows for “capture” of the maximum RV cavity, and provides a more direct assessment of the RV-pulmonary vascular axis between the RV free wall and the pulmonary circulation through the RV outflow tract.^10^ Although, in theory, the septum can be regarded as bi-layered and contributing to function of both ventricles, it is currently regarded as part of LV function.^53^

For optimal results, the same principles of image acquisition described for TD-derived deformation apply to 2D STE. Echocardiographic evaluation can be acquired in the resting state without sedation and gray scale images need to illustrate walls clearly and without artifact. An ECG signal is also mandatory in addition to event timing annotation, as described earlier. Fundamental and harmonic imaging with different probe types have not shown differences in strain and SR values in an in vitro study.^49^ The choice of transducer should be based on the frequency, which obtains the highest quality images with clear speckles. 2D STE is angle independent so the transducer can be manipulated off plane to obtain the ideal image. Sanchez et al. demonstrated that in order to obtain optimal reproducibility results for longitudinal strain assessment, an FR to HR ratio of at least 0.7–0.9 needs to be applied to the acquired images. Manipulations of depth and sector width can be used to achieve this ratio.^54^ Generally, an FR of 110–130 is required for preterm infants and 90–110 for term infants.^54^ The images should be optimized to demonstrate the speckles and endocardial and epicardial borders clearly.

In neonates, analysis of deformation parameters is performed offline using dedicated vendor-customized analysis software.^3^ Newer packages of imaging and software systems allow for strain measurements to be assessed on the ultrasound machine in real time during image acquisition, but its feasibility and reliability have not been assessed in neonates. Vendor-independent software packages are available for speckle tracking analysis with any image acquisition platform.^19^ In some software packages, an ROI is defined by tracing the endocardial border of the myocardium at end-systole. The width of the ROI is set to match the width of the wall of interest (Fig. 8). The software then automatically tracks the movement of speckles to derive the deformation parameters. The acceptability of the tracking is automatically suggested by the software. The user can also visually inspect the quality of the tracking before finally accepting or rejecting analysis of the segment. To enhance the STE capabilities, the ROI is readjusted repeatedly to avoid free wall base over excursion, tracking of the trabeculations, and avoid artifact. Cine-loop images with persistently inadequate tracking should be excluded from analysis. Once the integrity of myocardial STE is visually confirmed by the user, the software algorithm generates seven curves for each view of heart (i.e., apical four chamber, parasternal short axis view at the mitral valve, etc.) based on the timing of the opening and closure of the semilunar valves (aortic valve for LV strain and pulmonary valve for RV strain). Each curve represents the measured myocardial deformation (strain) for the six specific myocardial segments (basal septum, mid-septum, apical septum, basal lateral, mid-lateral, and apical lateral) and one global value representing the combined strain from all segments within the specific echocardiographic view (Fig. 10).

## Reference ranges and clinical utility in the neonatal population

Normative data and reference ranges are still emerging for deformation parameters obtained using 2D STE in the preterm and term infants. Reference ranges have been published in healthy uncomplicated term^10,35,37,45,51,55–58^ and preterm population,^11,19,26,32,53,59,60^ or reported results from control groups of neonates (who were recruited for specific studies; ^61–66^). The process of standardization and reference values in neonates stems from relatively small number of infants included in each study, the varying time points at which echocardiograms were acquired in the first year of age, and the multitude of vendors and software versions utilized for acquisition and post-processing. In addition, only two studies have assessed true “global” LV longitudinal (from the three apical chamber views; ^11,51^), while most have reported LV longitudinal strain values from the LV four-chamber.^10,19,53,57,58,64,65^ There are only two studies that report circumferential strain, and both only generate circumferential strain from the mid-ventricular (papillary muscle) level of the LV free wall.^60,64^ Table 3 summarizes the current available literature. Radial deformation values and diastolic SR parameters (early and atrial) measured using 2D STE remain unreliable in the neonatal population.^26^ In general, LV deformation parameters measured using 2D STE appear to remain stable during the transitional period and up to 28 days.^19,26,65–67^ RV strain parameters gradually increase beyond the transitional period and through the first year of age.^11,26,59^ Strain and SRs values are higher in the RV than the LV, reflective of the changing loading conditions specific to each ventricle.^11,26^ In the LV, circumferential deformation parameters appear to be slightly higher than longitudinal deformation.^60,64^

**Table 3:** Reference ranges for speckle tracking echocardiography deformation parameters in the neonatal population





Values presented as means and standard deviations if available, unless stated otherwise. All strain units are % and strain rate 1/s

*Gest* gestation, *N* number of infants, *Wt* birthweight, *Wks* weeks, *LV* left ventricle, *RV* right ventricle, *Sep* septum, *PMA* post-menstrual age, *Circ* circumferential, *LS* longitudinal strain, *SRs* systolic strain rate, *SRe* early diastolic strain rate, *SRa* late diastolic strain rate

2D STE has also been examined in some disease states in neonates.^2,27,61,63,66^ One of the first studies of 2D STE in preterm infants illustrated the negative impact of PDA ligation on LV GLS in the immediate post-operative period, followed by recovery 24 h later.^2^ The reduction in LV GLS post-operatively was attributed to the increase in afterload and the decrease in preload associated with the procedure. In the early transitional period, another study demonstrated that the administration of antenatal magnesium sulfate is associated with a lower SVR and a higher LV GLS on day 1 of age.^27^ These studies further highlight the load dependency of strain. The influence of common cardiopulmonary abnormalities in preterm infants (such as CLD and pulmonary hypertension) appear to leave a negative effect on RV and septal strain, with preservation of LV strain patterns.^11,61,65^ LV and RV function have also been evaluated in term infants of diabetic mothers (gestational and pre-gestational diabetes; ^63,68^). LV GLS is lower in pre-gestational (−10.4 ± 3.2, *n* = 20) and gestational (−13.1 ± 4.7, *n* = 25) groups when compared with the control group (−19 ± 2, *n* = 45) (*p* < 0.01).^63^ Similarly, LV GLS can identify dysfunction in severely asphyxiated term infants who are undergoing therapeutic hypothermia when compared with healthy controls (−11.01% ± 2.48 vs. −21.45% ± 2.74, *p* < 0.001; ^66^). LV GLS has a significant correlation with troponin levels (*r*^2^ = 0.64, *p* < 0.001) suggesting that LV GLS is also capable of grading disease severity.^66^ Finally, 2D STE strain was found to be significantly lower in term infants with proven sepsis in the first month of age when compared to age- and weight-matched controls.

## LV rotational mechanics

### Principles and validation in the neonatal population

**Fig. 11:**
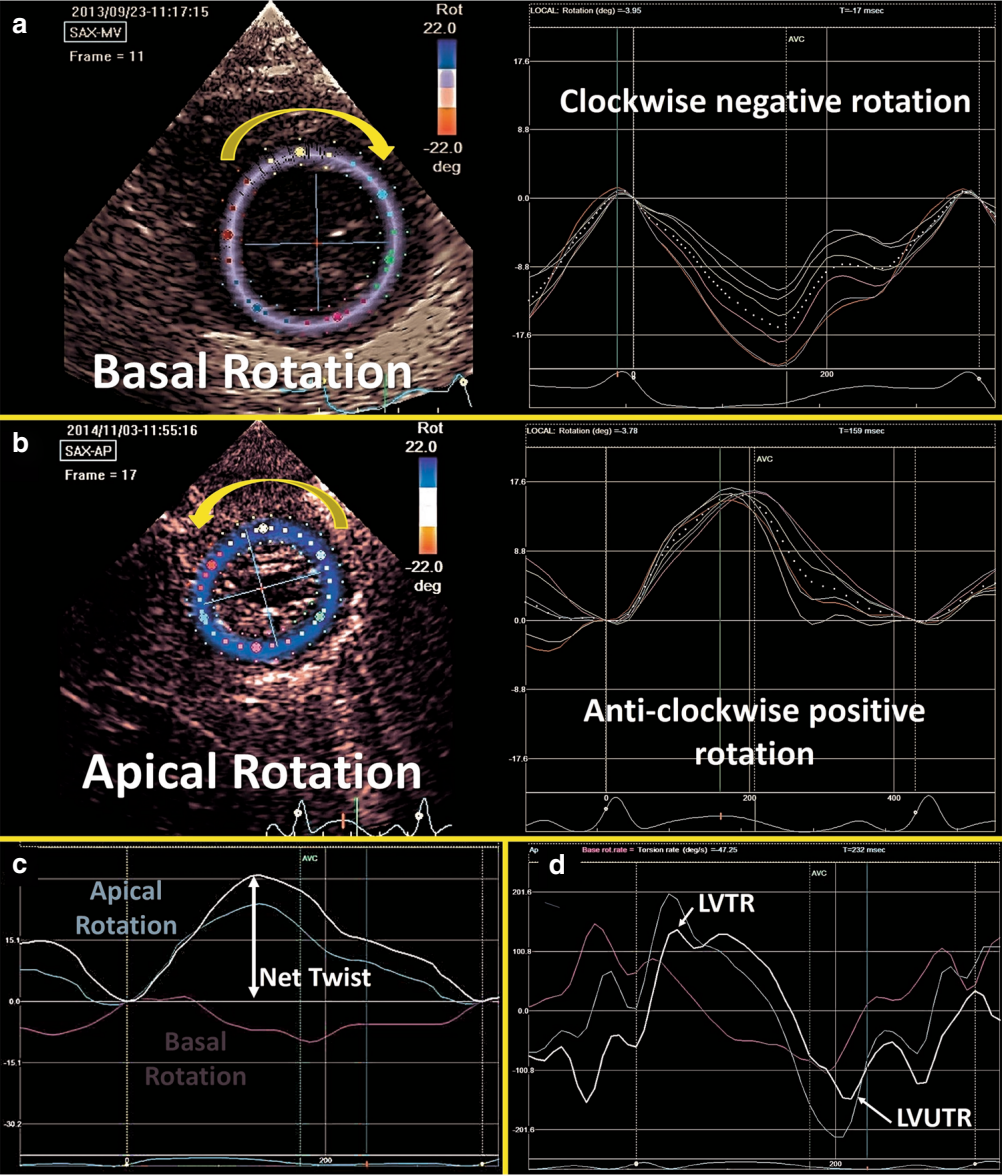
Left ventricle rotational mechanics. **a** Basal rotation occurs in a clockwise direction (negative) and **b** Apical rotation occurs in an anti-clockwise direction (positive). **c** The net effect of the opposing rotations is called Twist. **d** The speed at which twist occurs is called twist rate (LVTR) and the speed at which untwist occurs is called untwist rate (LVUTR)

Myocardial shear deformation in the circumferential-longitudinal plane results in torsional deformation of the LV during ejection and is utilized to characterize functional changes in systole and diastole.^69^ The complex architecture of the LV myocardium results in inhomogeneous contraction patterns. The myofiber orientation changes continuously from a right-handed helix in the subendocardium to a left-handed helix in subepicardium, enabling the LV to have unique rotational properties.^50^ The LV base rotates in a clockwise direction (displayed as negative rotation in degrees, Fig. 11a) and the apex rotates in a counter clockwise direction (displayed as positive rotation in degrees. Figure 11b; ^14^). Twist (degrees) is defined as the difference between apical and basal systolic rotation (Fig. 11c), and the net effect of this phenomenon is an LV wringing motion that improves the ability of the LV to eject blood during systole. Torsion (°/cm) is the term used to describe LV twist indexed to its LV end-diastolic length (LV length is measured from the apical four-chamber view in diastole by measuring the distance between the midpoint of the mitral valve annulus and the apex) and enables the comparison of LV twist across differing LV sizes. The temporal derivative of twist is referred to as twisting and untwisting rate (°/s; ^21^). LV twist rate (LVTR) is the velocity at which twist occurs per unit time and is depicted as a positive value (°/s). Untwisting is the motion opposite to the direction of twist occurring in diastole. The speed at which LV untwist occurs is termed LV untwist rate (LVUTR; Fig. 11d). During diastole, the LV untwists to return to its baseline un-deformed and untwisted shape. The act of untwisting also aids in diastolic function and contributes to early diastolic filling. This process is highly dependent on the elasticity of the LV.^70^ LV untwist is facilitated by the kinetic energy stored during twisting in systole and therefore, LVUTR is highly dependent on LVTR (an example of systolic–diastolic interdependence).^12^ Like natural strain, increasing afterload appears to decrease LV twist and untwist rate in experimental animal models and human adults.^71^ Similarly, in preterm infants increased afterload appears to negatively impact these measurements.^27^ LVUTR also appears to be negatively influenced by increasing afterload, as it is highly dependent on LV twist.^12^ Ramani et al. demonstrated that basal LV rotation was reduced with preserved LV apical rotation in adult patients with pulmonary arterial hypertension.^72^

There is a small, yet growing literature on the validation of rotational mechanics in neonates.^12,63,73–75^ Although the recommendations of European Association of Cardiovascular Imaging, EACVI, and the American Society of Echocardiography (ASE) task force to Standardize Deformation Imaging urge caution in the use of twist and torsion because both parameters are poorly defined in 2D echocardiography, recent work has demonstrated the improving feasibility and reproducibility analysis of rotational mechanics in neonates.^12,73–75^ In preterm infants, James et al. demonstrated acceptable intra- and inter-observer reliability with ICC ranging from 0.70 to 0.89.^12^ Zhang et al. demonstrated that inter-operator COV was ≤5%.^73^ Al-Naami does not report reproducibility statistics, but comments that imaging was feasible in all subjects.^75^ Kim et al. reported slightly lower feasibility at 80% with ICC >0.9 for all parameters.^74^

### Image acquisition and offline measurement

Two-dimensional STE method is also used to derive rotational parameters. The methodology for image acquisition and offline assessment is similar to deformation assessment described above. Two-dimensional grayscale images of the LV base (at the level of the mitral valve leaflets) and apex (distal to the papillary muscles) are acquired from LV parasternal short-axis view. Image acquisition at the two planes of interest is carried out to ensure that the LV cross-section is as circular as possible. A protocol for rotational mechanics imaging and post-processing data analysis exists for 2D STE in neonates.^12^

### Reference ranges and clinical utility in neonates

There is a paucity of data on LV rotational physiology in neonates. There are several age-related studies that have assessed the changes in rotational mechanics from birth through early adolescents, which also include a small subset of neonates.^73–76^ There are a few single-study reports that define control cohorts to compare with specific diseases in term neonates.^63,68,77,78^ Al-Biltagi et al.^63^ showed that cardiac torsion was impaired in infants of diabetic mothers (IDMs), and Liao et al.^68^ demonstrated a similar decrease in torsion, but with persevered EF, suggesting that rotational mechanics may offer a more sensitive measure of ventricular function. Xie et al. evaluated the utility of torsion analysis in the assessment of infants with congenital heart disease.^77^

Rotational mechanics was recently studied in late preterm [mean (SD) gestation of 36.0 (±1.5), *n* = 31]^27^ and extreme preterm infants [mean (SD) gestation of 26.8 weeks (±1.5), *n* = 51].^12^ In extreme preterm infants, apical rotation remains constant over the first week of age [11.8° (±5.0) vs. 12.1° (±6.1) vs. 11.7° (±8.3), on days 1, 2, and 5–7; *p* = 0.92]. Basal rotation, however, changes from counter clockwise on day 1 and 2 to clockwise on day 7 [5.5 [−0.3 to 8.3] vs. 4.0 [−4.7 to 7.2] vs. −4.5 [−5.8 to −2.3], *p* < 0.001] with a resultant net increase in twist, torsion, and LVUTR.^12^ Future studies are now required to determine the clinical relevance of rotational mechanics, the effects of various disease states (cardiomyopathies, chronic cardio-respiratory disease, sepsis, etc.), explore the relationships to conventional echocardiographic measures, and to determine reference ranges in preterm and term infants.

## Conclusion

The assessment of deformation parameters to properly characterize cardiac function in neonates has gained considerable interest. The emerging literature continues to demonstrate the feasibility and reproducibility of strain values derived by both TDI and 2D STE in the neonatal (term and preterm) population, and the relative advantages of those techniques when compared to conventional measures. With the establishment of reference ranges and normative data, the routine clinical use of strain, SR, and rotational mechanics is likely to become more common. As the clinical applicability of these measures is further elucidated in neonates, we will begin to understand their ability to direct management, monitor treatment response, and predict outcomes to optimize the care delivered to neonates. Future work should focus on the ability of those measurements to distinguish between myocardial dysfunction secondary to adverse loading conditions and dysfunction resulting from impaired intrinsic contractility (or a mixture of both). This will help tailor the therapeutic interventions to more accurately target the underlying pathophysiological consequences of disease states.

## Appendix

**European Special Interest Group ‘Neonatologist Performed Echocardiography’ (NPE), endorsed by the European Society for Paediatric Research (ESPR) and European Board of Neonatology (EBN)**

**de Boode W. P.** (chairman), Department of Neonatology, Radboud University Medical Center, Radboud Institute for Health Sciences, Amalia Children’s Hospital, Nijmegen, The Netherlands (willem.deboode@radboudumc.nl)

**Austin T.**, Department of Neonatology, Rosie Hospital, Cambridge University Hospitals NHS Foundation Trust, Cambridge, United Kingdom (topun.austin@addenbrookes.nhs.uk)

**Bohlin K.**, Department of Neonatology, Karolinska University Hospital, Karolinska Institutet, Stockholm, Sweden (kajsa.bohlin@ki.se)

**Bravo M. C.**, Department of Neonatology, La Paz University Hospital, Madrid, Spain (mcarmen.bravo@salud.madrid.org)

**Breatnach C. R.**, Department of Neonatology, The Rotunda Hospital, Dublin, Ireland (colm.breatnach@gmail.com)

**Breindahl M.**, Karolinska University Hospital, Karolinska Institutet, Stockholm, Sweden (morten.breindahl@sll.se)

**Dempsey E.**, INFANT Centre, Cork University Maternity Hospital, University College Cork, Ireland (g.dempsey@ucc.ie)

**El-Khuffash A.**, Department of Neonatology, The Rotunda Hospital, Dublin, Ireland; Department of Pediatrics, The Royal College of Surgeons in Ireland, Dublin, Ireland (afifelkhuffash@rcsi.ie)

**Groves A. M.**, Division of Newborn Medicine, Mount Sinai Kravis Children’s Hospital, New York, NY, USA (alan.groves@mssm.edu)

**Gupta S.**, University Hospital of North Tees, Durham University, Stockton-on-Tees, United Kingdom (samir.gupta@nth.nhs.uk)

**Horsberg Eriksen B.**, Department of Pediatrics, Møre and Romsdal Hospital Trust, Ålesund, Norway (beate.eriksen@me.com)

**Levy P. T.**, Department of Pediatrics, Washington University School of Medicine, Saint Louis, MO, USA; Department of Pediatrics, Goryeb Children’s Hospital, Morristown, NJ, USA (Levy_P@kids.wustl.edu)

**McNamara P. J.**, Departments of Pediatrics and Physiology, University of Toronto, Toronto, ON, Canada (patrick.mcnamara@sickkids.ca)

**Molnar Z.**, John Radcliffe Hospital, Oxford, United Kingdom (zoltan.Molnar@ouh.nhs.uk)

**Nestaas E.**, Institute of Clinical Medicine, Faculty of Medicine, University of Oslo, Norway; Department of Cardiology and Center for Cardiological Innovation, Oslo University Hospital, Rikshospitalet, Oslo, Norway; Department of Paediatrics, Vestfold Hospital Trust, Tønsberg, Norway (nestaas@hotmail.com)

**Rogerson S. R.**, The Royal Women's Hospital, Parkville, VIC, Australia (sheryle.Rogerson@thewomens.org.au)

**Roehr C. C.**, Department of Paediatrics, University of Oxford, John Radcliffe Hospital, Oxford, United Kingdom (charles.roehr@paediatrics.ox.ac.uk)

**Savoia M**, Azienda Ospedaliero-Universitaria S. Maria della Misericordia, Udine, Italy (marilena.savoia@gmail.com)

**Schubert U.**, Department of Clinical Science, Intervention and Technology, Karolinska Institutet, Stockholm, Sweden (ulfschubert@gmx.de)

**Schwarz C. E.**, Department of Neonatology, University Children’s Hospital of Tübingen, Tübingen, Germany (c.schwarz@med.uni-tuebingen.de)

**Sehgal A.**, Department of Pediatrics, Monash University, Melbourne, Australia (arvind.sehgal@monash.edu)

**Singh Y.**, Addenbrooke's Hospital, Cambridge University Hospitals NHS Foundation Trust, Cambridge, United Kingdom (yogen.Singh@nhs.net)

**Slieker M. G.**, Department of Paediatric Cardiology, Radboudumc Amalia Children’s Hospital, Nijmegen, The Netherlands (Martijn.Slieker@radboudumc.nl)

**Tissot C.**, Department of Pediatrics, Clinique des Grangettes, Chêne Bougeries, Switzerland (cecile.tissot@hotmail.com)

**van der Lee R.**, Department of Neonatology, Radboud University Medical Center, Radboud Institute for Health Sciences, Amalia Children’s Hospital, Nijmegen, The Netherlands (Robin.vanderLee@radboudumc.nl)

**van Laere D.**, Department of Pediatrics, Antwerp University Hospital UZA, Edegem, Belgium (david.VanLaere@uza.be)

**van Overmeire B.**, Department of Neonatology, University Hospital Brussels, Brussels, Belgium (bart.van.overmeire@erasme.ulb.ac.be)

**van Wyk L.**, Department of Paediatrics & Child Health, University of Stellenbosch, Cape Town, South Africa (lizelle@sun.ac.za)
